# A disulfide chaperone knockout facilitates spin labeling and pulse EPR spectroscopy of outer membrane transporters

**DOI:** 10.1002/pro.4704

**Published:** 2023-07-01

**Authors:** Viranga W. Wimalasiri, Kinga A. Jurczak, Monika K. Wieliniec, Thushani D. Nilaweera, Robert K. Nakamoto, David S. Cafiso

**Affiliations:** ^1^ Department of Chemistry and Center for Membrane Biology University of Virginia Charlottesville Virginia USA; ^2^ Department of Molecular Physiology and Biological Physics University of Virginia Charlottesville Virginia USA; ^3^ Present address: Genetics and Biochemistry Branch National Institute of Diabetes and Digestive and Kidney Diseases Bethesda Maryland USA

**Keywords:** in vivo EPR spectroscopy, outer membrane proteins, site‐directed spin‐labeling, TonB‐dependent transport

## Abstract

Pulse EPR measurements provide information on distances and distance distributions in proteins but require the incorporation of pairs of spin labels that are usually attached to engineered cysteine residues. In previous work, we demonstrated that efficient in vivo labeling of the *Escherichia coli* outer membrane vitamin B_12_ transporter, BtuB, could only be achieved using strains defective in the periplasmic disulfide bond formation (Dsb) system. Here, we extend these in vivo measurements to FecA, the *E. coli* ferric citrate transporter. As seen for BtuB, pairs of cysteines cannot be labeled when the protein is present in a standard expression strain. However, incorporating plasmids that permit an arabinose induced expression of FecA into a strain defective in the thiol disulfide oxidoreductase, DsbA, enables efficient spin‐labeling and pulse EPR of FecA in cells. A comparison of the measurements made on FecA in cells with measurements made in reconstituted phospholipid bilayers suggests that the cellular environment alters the behavior of the extracellular loops of FecA. In addition to these in situ EPR measurements, the use of a DsbA minus strain for the expression of BtuB improves the EPR signals and pulse EPR data obtained in vitro from BtuB that is labeled, purified, and reconstituted into phospholipid bilayers. The in vitro data also indicate the presence of intermolecular BtuB‐BtuB interactions, which had not previously been observed in a reconstituted bilayer system. This result suggests that in vitro EPR measurements on other outer membrane proteins would benefit from protein expression in a DsbA minus strain.

## 1 INTRODUCTION

Active transport in the outer membrane (OM) of Gram‐negative bacteria is facilitated by a family of proteins that bind to and obtain energy from the inner membrane (IM) protein TonB (Noinaj et al., 2010). These TonB‐dependent transporters (TBDTs) are responsible for the acquisition of trace nutrients, such as iron, nickel, and vitamin B_12_, as well as the uptake of carbohydrates. TonB‐dependent transporters are critical for the success of many pathogens (Braun, 2001; Klebba et al., 2021), and they are essential for the proper functioning of the human microbiome (Bolam & van den Berg, 2018). Although numerous crystal structures have been obtained for this family of transporters (Pollet et al., 2021), the molecular mechanisms by which they function are poorly understood (Ratliff et al., 2021).

Site‐directed spin labeling when combined with EPR spectroscopy is a powerful and sensitive method that can reveal structural changes and conformational exchange in membrane transport proteins (Hubbell et al., 2013). This approach has been used to examine structural transitions and conformational exchange in BtuB, the *Escherichia coli* TonB‐dependent vitamin B_12_ transporter (Fanucci et al., 2003; Xu et al., 2006), as well as other *E. coli* TBDTs such as the ferric citrate transporter, FecA (Kim et al., 2007; Mokdad et al., 2012), the ferrichrome transporter FhuA (Sarver et al., 2018), and the ferric enterobactin receptor, FepA (Jiang et al., 1997; Klug et al., 1998). Most of this work has been performed in vitro on detergent purified and membrane reconstituted protein. Because transport requires an interaction between the transporter in the OM and TonB in the IM, transport has never been reconstituted, and one issue with the in vitro experiments on BtuB and other TBDTs is that the function of the transporter has never been established.

It is possible to perform continuous wave (CW) EPR on TBDTs in vivo (Jiang et al., 1997), and more recently pulse EPR measurements have been carried out in situ on BtuB when it is over‐expressed in intact bacteria (Joseph et al., 2019). Measurements on the intact cell have the advantage that they can be carried out on a transport protein that is known to be functional and where it is present in its native environment. Remarkably, this outer membrane environment appears to modify protein structure and behavior. For example, in isolated OM preparations (Sikora et al., 2016) or reconstituted phospholipid bilayers (Kim et al., 2008), the extracellular loops of BtuB sample a wide range of conformations in the apo state and undergo a gating motion with substrate addition; however, in the cell, loop conformations are restricted, and substrate is not observed to modulate the extracellular loops (Nyenhuis, Nilaweera, & Cafiso, 2020). Different behaviors are also observed in the core region of BtuB. For example, structural changes in the core region of the protein that could mediate transport are observed in the cell but not observed in reconstituted phospholipid membranes (Nilaweera et al., 2021). Compared to measurements made in cells, measurements in purified reconstituted systems do have the advantage that they generally yield spectra with better signal to noise and lower levels of nonspecific labeling. They also enable the characterization of protein–protein interactions between BtuB and fragments of the inner‐membrane machinery (Freed et al., 2013).

Pulse EPR experiments, such as double electron–electron resonance (DEER), provide information on the distances and distance distributions between pairs of spin labels (Jeschke, 2012). This is most easily performed on proteins through the covalent modification of pairs of reactive cysteine residues that have been engineered into the protein. In intact *E. coli*, outer membrane proteins typically have zero or an even number of cysteines (Dutton et al., 2008). BtuB lacks native cysteines and single cysteines that are incorporated into BtuB can be efficiently spin labeled in vivo using the K12‐derived strain RK5016 (Nilaweera et al., 2019). However, when pairs of cysteines are incorporated into BtuB and the protein is expressed in RK5016 under conditions where the cell viability is maintained, in vivo labeling of BtuB does not occur, and this is a direct consequence of the presence of a periplasmic disulfide oxidation (Dsb) system that functions to cross‐link pairs of cysteines (Landeta et al., 2018). Labeling can be performed on pairs of cysteines in BtuB in vivo using an *E. coli* strain deficient in the activity of either DsbA or DsbB (Nilaweera et al., 2019).

In the present work we show that FecA, the *E. coli* ferric citrate transporter, cannot be double spin‐labeled in vivo when expressed in the commonly used BL21(DE3) strain, but can be efficiently double‐labeled when it is expressed in a DsbA minus strain. Excellent CW and pulse EPR data are obtained from FecA in cells with minimal background labeling. The initial data suggest that extracellular loop 8, which is seen to undergo large movements by crystallography upon substrate binding (Yue et al., 2003), samples two states in cells with substrate addition, suggesting that the environment used for crystallography has trapped one substate. For in vitro preparations of BtuB, where the protein is purified and reconstituted into phospholipid vesicles, the use of a DsbA minus strain enhances overall labeling efficiency, indicating that double spin labeling even on the purified isolate protein is improved using a Dsb deficient strain. Evidence for BtuB‐BtuB interactions that had previously been seen only in cells is seen in membrane reconstituted BtuB that is obtained from the DsbA minus strain, and this is likely a result of the enhanced labeling efficiency. Work comparing the results obtained in cells with data obtained in a purified phospholipid reconstituted membrane indicates that FecA, like BtuB, behaves differently in the cell than it does in the purified reconstituted environment.

## 2 RESULTS

### 2.1 Expression in dsbA ^−^ strain improves the labeling efficiency of BtuB in vitro

**FIGURE 1:**
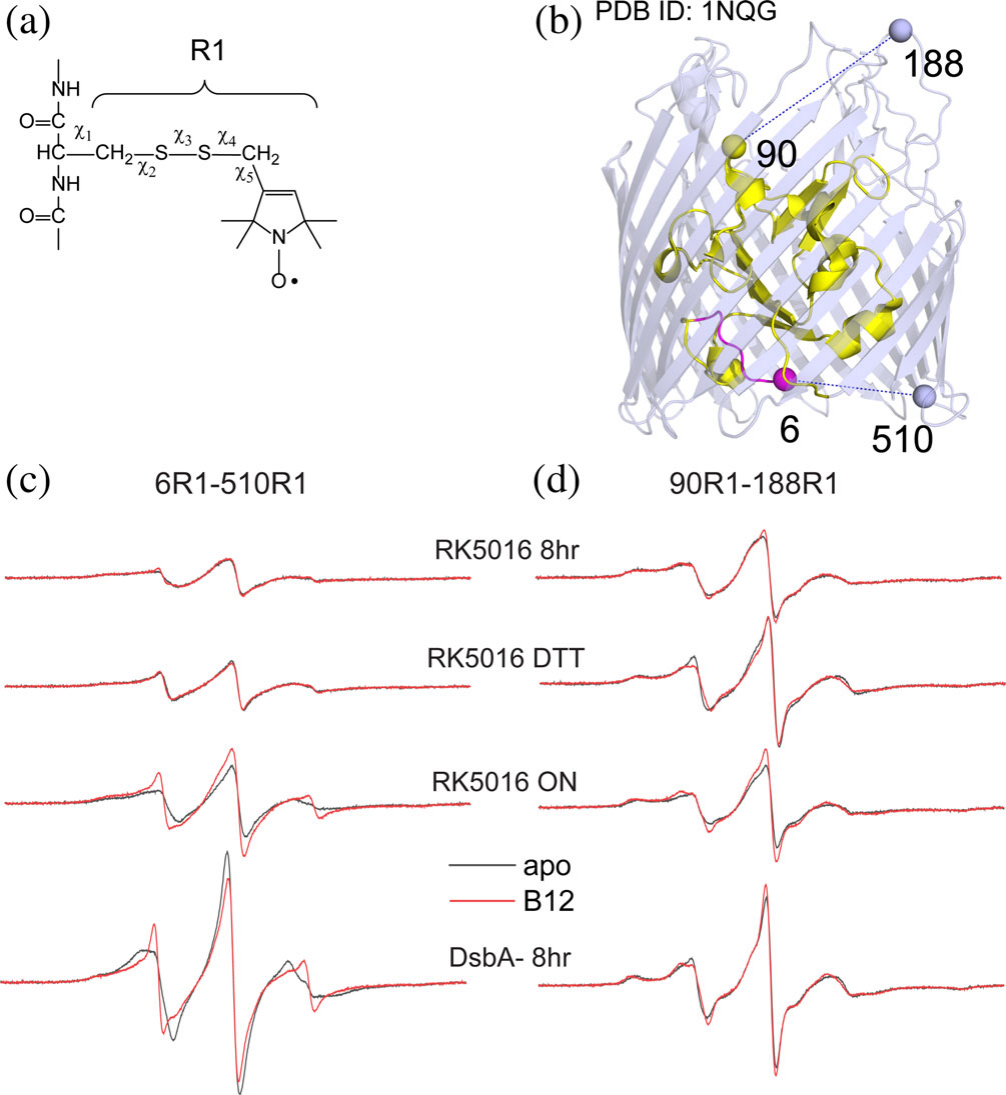
The use of DsbA deficient strain improves the relative labeling efficiency of BtuB in a phospholipid reconstituted preparation. (a) For EPR measurements, free cysteines are labeled using a conventional MTSL spin label to generate the side chain R1. In (b) crystal structure of BtuB (PDB ID: 1NQG) with sites labeled on the extracellular and periplasmic surfaces of BtuB shown as spheres. The periplasmic energy coupling Ton box is shown in magenta. In (c) are shown the EPR spectra obtained from 6R1‐510R1 on the periplasmic interface where BtuB is over‐expressed in either the RK5016 strain or a *dsbA* ^*−*^ strain. The RK5016 strain was grown either for 8 h or overnight (ON). In one case BtuB was taken through an initial ion‐exchange purification and was subjected to DTT treatment and a buffer exchange. The protein was then spin labeled and taken through a second ion‐exchange purification prior to reconstitution. Each reconstituted preparation includes the same quantity of BtuB and POPC. In (d) are shown the EPR spectra for the 90R1‐188R1 spin pair for BtuB that was expressed under the same conditions as shown in (c).

Figure 1 shows a series of EPR spectra obtained from phospholipid reconstituted BtuB at two pairs of sites, D6R1‐Q510R1 on the periplasmic surface (Figure 1c) and V90R1‐T188R1 on the extracellular surface (Figure 1d). The reconstituted BtuB proteoliposomes from which these spectra are obtained all contain equivalent levels of protein, where the reconstitution is performed in an identical manner. As indicated, BtuB was labeled and purified from *E. coli* preparations that were grown for different time periods and under different conditions in the RK5016 strain; BtuB was also labeled and purified from a strain where the thiol disulfide oxidoreductase, DsbA, was not functional. As a result, the differences in signal intensity seen in Figure 1 reflect differences in labeling efficiency by the MTSL reagent.

For the D6R1‐Q510R1 pair (Figure 1c) the 8‐h growth (top trace) yields relatively weak EPR signals that undergo an increase in amplitude of about 30% when the sample is treated with 6 mM DTT treatment prior to labeling. Site 6 in BtuB is located in the N‐terminal Ton box, an energy coupling segment that is known from previous in vitro work to undergo a substrate‐induced unfolding (Fanucci et al., 2003; Freed et al., 2010; Xu et al., 2006); however, the slight improvement in signal intensity with DTT treatment fails to yield clear evidence for a substrate‐induced unfolding of the Ton box (apo and B_12_‐bound traces are in gray and red, respectively). This earlier work was performed on *E. coli* that were grown for an extended time, and we repeated the measurement using BtuB produced from cells grown overnight. With an overnight growth, the EPR signal amplitudes are increased and the substrate‐induced change in the Ton box is now apparent in the EPR spectrum. However, if the cells are grown for 8 h in the *dsbA* ^*−*^ strain, so that pairs of cysteines are not enzymatically oxidized in the periplasm by the Dsb system (Landeta et al., 2018), there is an approximately 6‐fold increase in the amplitude of the resulting EPR signals relative to the 8 h growth once the protein is purified and reconstituted. The EPR signals from the V90R1‐T188R1 spin pair (Figure 1d) are also improved upon expression in the *dsbA* ^*−*^ strain, although the increase in signal intensity is not as dramatic, in this case showing a 2‐fold enhancement in signal intensity relative to equivalent levels of protein obtained from the standard RK5016 strain.

In addition to facilitating the labeling of BtuB in intact *E. coli* (Nilaweera et al., 2019), the data in Figure 1 indicate that the Dsb system impacts the labeling of purified and membrane reconstituted BtuB. We speculate that the improved signal intensities observed when the growth time for the RK5016 strain is extended is due to the culture entering a stationary phase, where cell growth is balanced by cell death and lysis. Cell lysis produces conditions that are reducing, and this may act to reduce oxidized cysteine pairs and increase their reactivity toward the MTSL reagent. Extended cell growth times and expression of BtuB may also compromise the Dsb system and allow some cysteine pairs to escape oxidation in the periplasm.

### 2.2 Expression in a dsbA ^−^ strain improves pulse EPR signals for labeled BtuB in both reconstituted and OM preparations

**FIGURE 2:**
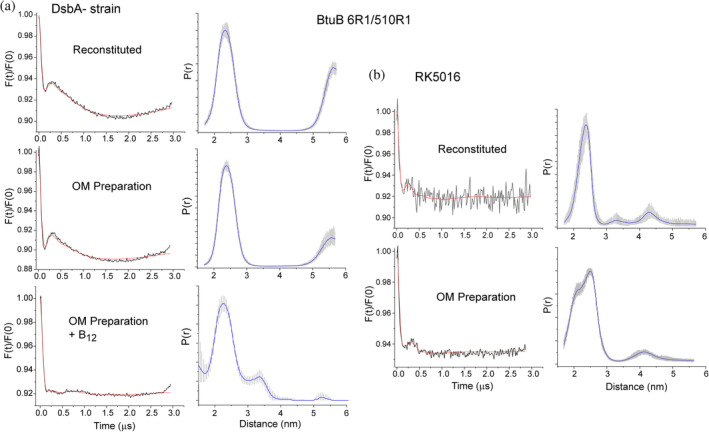
DEER data from purified reconstituted protein or an outer‐membrane preparation are improved using a DsbA deficient strain. In (a) DEER data obtained for BtuB labeled at sites D6 and Q510 when isolated from a *dsbA* ^*−*^ strain following an 8 h growth and reconstituted into POPC bilayers or obtained from an OM preparation. Data are shown in the apo state for BtuB in the reconstituted state (top), from the OM preparation (middle) and in the OM preparation following vitamin B_12_ addition (bottom). In (b) DEER data obtained from BtuB produced from an 8 h growth in RK5016 when labeled at sites 6 and 510 in either the reconstituted vesicle preparation or OM preparation in the apo state. All distance distributions were obtained using DEERNet, except for the bottom panel in (a) where Tikhonov regularization and a validation procedure were used (see Section 4).

In addition to improving the quality of the CW EPR spectra obtained from purified BtuB, the use of the DsbA deficient strain also improves DEER signals obtained from reconstituted or OM preparations. Shown in Figure 2a are examples of DEER data obtained using the *dsbA* ^*−*^ strain for the D6R1‐Q510R1 spin pair. Both the purified as well as the OM preparation yield similar dipolar evolutions and similar distance distributions.

In these corrected DEER data, there appears to be a long but poorly defined long‐distance component. This component resembles an intermolecular component that was observed previously in cells (Nyenhuis, Nilaweera, Niblo, et al., 2020), although the exact position and width of this longer distance is not well defined due to the relatively short echo times in these experiments. In this previous work, this long‐distance component was consistent with the formation of strings of BtuB monomers that are thought to drive the formation of OMP islands (Rassam et al., 2015). This feature had not previously been observed in reconstituted systems where protein was expressed from a strain with an intact Dsb system. As shown in Figure 2b, when expressed with a functional Dsb system, reconstituted or OM samples of labeled BtuB are noticeably poorer than those lacking an active Dsb system.

### 2.3 The use of a DsbA deficient strain facilitates double spin‐labeling of FecA in vivo

**FIGURE 3:**
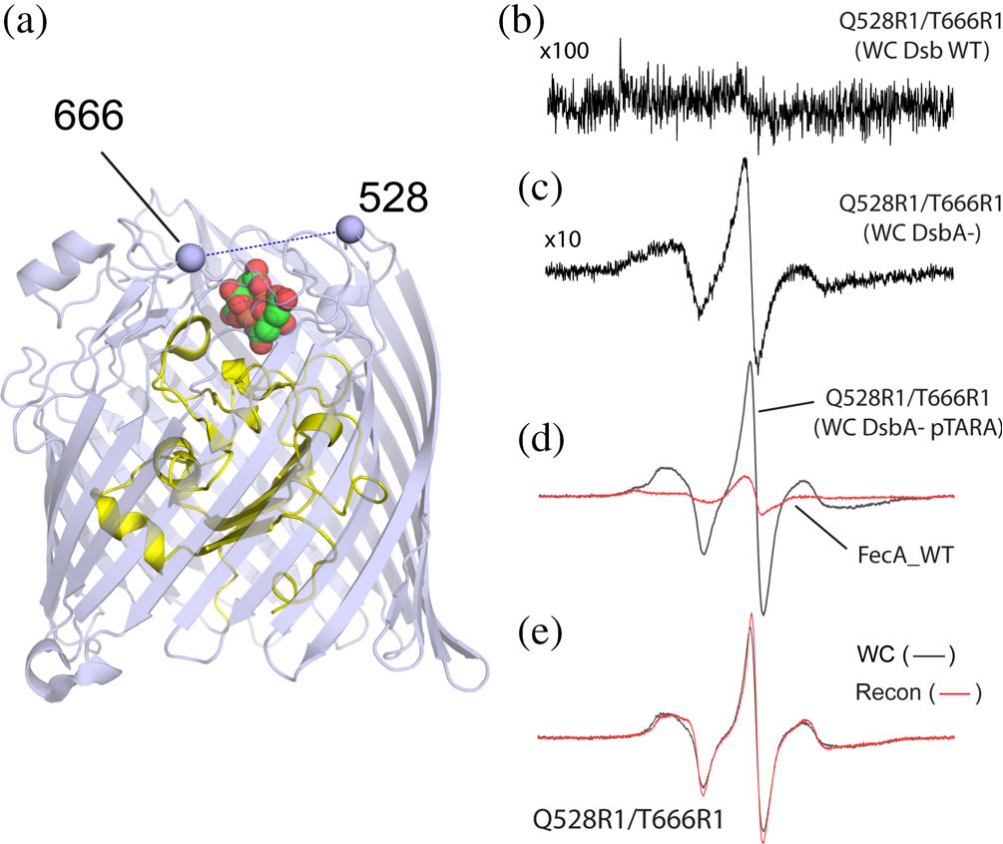
Double spin labeling of FecA in vivo requires a DsbA deficient strain. Shown in (a) crystal structure of FecA bound to ferric citrate (PDB ID: 1KMP) with the Cα carbons of sites 528 (loop 8) and 666 (loop 11) shown as spheres. In (b) in vivo EPR spectrum obtained using strain RI89 with an intact Dsb system; and (c) EPR spectrum obtained from a *dsbA* ^*−*^ strain lacking induction by T7 polymerase. The relative receiver gains used in (b) and (c) are indicated. In (d) is the EPR spectrum obtained in vivo for Q528R1/T666R1 using the *dsbA* ^*−*^ strain RI90 along with the pTARA plasmid and arabinose induction of the T7 polymerase. The background signal obtained when WT FecA is expressed is also shown (red trace). Shown in (e) is a comparison of the EPR spectra obtained from Q528R1/T666R1 in vivo and in a reconstituted preparation. In (e), the background signal has been subtracted from the in vivo spectrum.

As indicated above, we demonstrated previously that BtuB could not be efficiently double labeled in vivo when expressed in the K12‐deived RK5016 strain (Nilaweera et al., 2019). For the iron transporter, FecA, attempts to label the protein when produced in BL21(DE3) or in RI89 (with a functional Dsb system) failed to produce a measurable spectrum in vivo. Shown in Figure 3 are EPR spectra obtained from FecA where we attempted to label two sites (Q528C and T666C) in the 8–11 extracellular loops of FecA (Figure 3a). Shown in Figure 3b is the EPR spectrum obtained when we attempted to label the Q528C‐T666C cysteine pair in vivo using a strain with an active Dsb system (RI89). As seen, essentially no signal can be detected. When we repeated the labeling using the *dsbA* ^*−*^ strain having an inactive DsbA system, a measurable signal was obtained and is shown in Figure 3c. Although FecA is under the control of a T7 promoter and requires the T7 polymerase (which is not present in the *dsbA* ^*−*^ strain), there is apparently enough expression of FecA to allow some labeling and the acquisition of an EPR spectrum. However, when the T7 polymerase is expressed from the pTARA plasmid upon addition of arabinose, the labeling of FecA is dramatically elevated and a relatively noise‐free EPR spectrum is obtained (Figure 3d). The level of labeled FecA in cells is clearly improved over the case where the T7 polymerase is not being induced (Figure 3c) and obviously much better than the strain having a functional Dsb system (Figure 3b). Also shown on the same scale is the background signal obtained when WT FecA (lacking any cysteines) is expressed (red trace). A comparison between the Q666R1‐T666R1 spectra obtained in a purified reconstituted system versus the intact cell is shown in Figure 3e. In this case, there are minor differences in the spectra, but the motion of the labels and hence the environment around the labels in these two preparations is very similar.

### 2.4 DEER signals from FecA in situ reveal the presence of conformational gating in an extracellular loop

**FIGURE 4:**
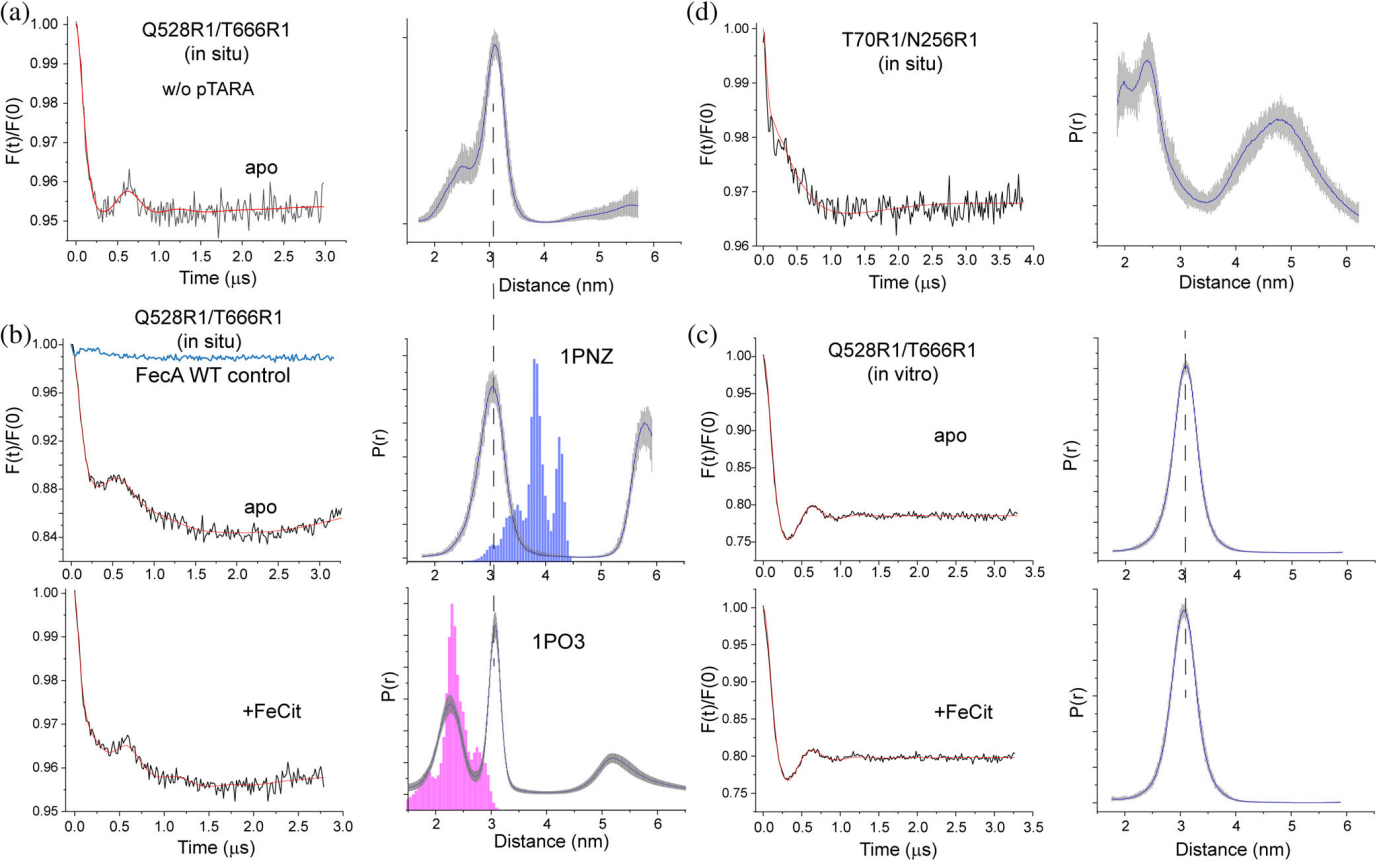
Conformational gating in the 8/11 extracellular loops is dependent upon environment. Shown in (a) is a DEER trace obtained in cells for the Q528C‐T666C double mutant of FecA in the *dsbA* ^*−*^ strain without induction by the pTARA plasmid. Shown in (b) are DEER traces in the apo and ferric citrate bound states for the Q528R1‐T666R1 spin pair when FecA expression in the *dsbA* ^*−*^ strain is induced by arabinose using the pTARA plasmid. Also shown is a DEER trace (blue line) obtained when WT FecA is expressed, and cells are taken though the same spin labeling procedure. One primary distance near 3 nm is seen in the apo state, but a second state at a shorter distance appears with ferric citrate addition. The predictions from the apo and substrate bound crystal structures (PDB ID: 1PNZ and 1PO3, respectively) are shown as blue and magenta histograms. In (c) are the apo and ferric citrate bound states of the 8/11 loops for the purified protein obtained from the *dsbA* ^*−*^ strain with the pTARA plasmid reconstituted into POPC vesicles. Shown in (d) are data from the periplasmic surface of FecA in cells where one label is in the Ton box (site 70), and a second is located on the periplasmic surface of the barrel (site 256).

Without inducing the expression of FecA, a measurable signal was obtained from the cell preparation (Figure 3c) and we were able to run pulse EPR on this sample and acquire a dipolar evolution (DEER signal) for FecA in the *dsbA* ^*−*^ strain. The data are shown in Figure 4a and yield a major distance component near 3.1 nm for the Q528R1‐T666R1 spin pair. However, without the induction FecA expression, the levels of labeled FecA are low, the dipolar DEER signal is noisy, and the data required an acquisition period of 24 h.

The data are dramatically improved when FecA expression is induced by expression of T7 polymerase using the pTARA plasmid. Shown in Figure 4b are DEER data obtained for FecA Q528R1‐T666R1 in the apo and substrate bound forms when FecA expression is induced by arabinose. As noted above (Figure 3d, red trace) there is some background labeling when WT FecA lacking any cysteines is induced and labeled in the *dsbA* ^*−*^ strain; however, this background signal fails to produce a measurable dipolar signal (Figure 4b, top panel, blue trace).

As seen in Figure 4b, in the apo state one well‐defined peak near 3 nm is obtained that also shows up in both the apo as well as the substrate bound forms. There is also a low frequency component in the background corrected DEER signal that may be due to a long‐distance component (in the 5–6 nm range). It is poorly resolved in terms of width and position but it resembles the intermolecular peak seen previously for BtuB in intact cells (Nyenhuis, Nilaweera, Niblo, et al., 2020).

The predicted distance distributions from the apo and ferric citrate bound crystal structures are shown in Figure 4b (blue and magenta histograms, respectively). In the apo state, there are distance components in the prediction arising from label rotamers that overlap with the experimental distance at 3.1 nm, but the center of the distribution is approximately 8 or 9 Å longer than the experimental distance. For the ferric citrate bound state, the crystal structure predicts a shorter distance near 2.4 nm, and the center of that distribution agrees well with experimentally measured distance. However, the original distance component at 3 nm is still present in the substrate bound form. Unlike the crystal structure where there is a well‐defined state for the 8–11 loops in the substrate bound state, these data suggest that both more open and closed states are simultaneously present in the substrate bound form. Although more work is needed to examine the loops in FecA, this result indicates that there may be an equilibrium between these conformations in the ligand bound state.

The use of the *dsbA* ^*−*^ strain also improved the data obtained from reconstituted samples, and Figure 4c shows data obtained from FecA (Q528R1‐T666R1) expressed in the *dsbA* ^*−*^ strain with arabinose induction using pTARA and purified in detergent by ion exchange. There are some notable differences in the in vitro data compared with the whole cell (in situ) sample. Although the peak near 3 nm is present in the reconstituted preparation in the apo state, there is no evidence for a movement or gating of the 8–11 loops with the addition of substrate (Figure 4c). In addition, there is no evidence for a long‐distance component, which also appears to be absent from the signal obtained in cells without the induction of FecA expression (Figure 4a).

Finally, as was the case for BtuB (Nilaweera et al., 2019), the *dsbA* ^*−*^ strain appears to facilitate the labeling of the periplasmic surface of FecA in cells (Figure 4d). The data are of noticeably poorer quality than that from the extracellular surface, but we expect that deuteration of the sample will improve data quality and permit an examination of the position of the FecA Ton box and transcriptional regulatory domain in cells.

## 3 DISCUSSION

The efficient in vivo spin labeling of pairs of cysteine residues in the vitamin B_12_ transporter, BtuB, was previously shown to require the use of an *E. coli* strain deficient in the thiol disulfide oxidoreductase, DsbA, or the DsbA oxidase, DsbB (Nilaweera et al., 2019). In the present work, we demonstrate that a strain deficient in DsbA activity is also necessary to double‐label cysteines in the extracellular loops of the *E. coli* ferric citrate transporter, FecA, in cells. Moreover, we show that in vitro EPR measurements on BtuB that is purified and reconstituted into bilayers is dramatically improved by the production of the protein in a *dsbA* ^*−*^ strain. We expect that both in vivo and in vitro labeling of pairs of cysteines may either require or be improved by the use of a *dsbA* ^*−*^ strain for a range of outer‐membrane proteins.

In previous work, it was possible to incorporate pairs of spin labels into BtuB and FecA in purified, reconstituted preparations (Mokdad et al., 2012; Xu et al., 2006), and in outer membrane preparations (Sikora et al., 2016) using conventional *E. coli* K12‐derived strains having a functional Dsb system. But often, obtaining adequate DEER data required some additional manipulations of the sample. For example, our first attempts to incorporate pairs of cysteines into BtuB (Xu et al., 2006) required a protocol for labeling that involved two ion‐exchange purifications with a DTT treatment after the first purification prior to spin labeling. The dramatic improvement seen here for the labeling of sites 6 and 510 on the periplasmic interface of BtuB when the *dsbA* ^*−*^ strain is used (Figure 1c) suggests that even in the purified, reconstituted system some significant level of internally disulfide cross‐linked BtuB is present when a wild‐type strain is use for protein production. As seen in Figure 1c, performing an overnight growth combined with DTT treatment improves the signal, but not to the same extent seen by the use of the *dsbA* ^*−*^ strain. It should be noted that the signal improvement obtained in vitro using the *dsbA* ^*−*^ strain was greatest for the pair of sites tested on the periplasmic surface of BtuB (Figure 1c). There was less improvement for the pair of sites on extracellular surface (Figure 1d). We do not presently understand the basis for this difference, but it may reflect the ability of the Dsb system to oxidize certain pairs of cysteine residues, perhaps because of the local protein dynamics or structure at these sites.

In previous whole cell measurements on BtuB, we demonstrated that long‐distances that appear in the distance distribution around 6 nm have an intermolecular origin (Nyenhuis, Nilaweera, Niblo, et al., 2020), and are likely due to specific BtuB‐BtuB interactions. These interactions are the basis for the formation of string‐like oligomers termed OMP islands (Rassam et al., 2015), which play a role in the turnover and movement of outer‐membrane proteins to the bacterial poles. Previously, we found no evidence for these intermolecular BtuB interactions in reconstituted systems, but they appear to be present in a reconstituted system (Figure 2a), when BtuB is expressed in a *dsbA* ^*−*^ strain. This observation is consistent with the idea that with an intact Dsb system, a significant level of protein is internally cross‐linked; and as a result, the protein population may not be well‐labeled. In the intact cell, the long distances observed for BtuB were consistent with the presence of a single LPS molecule interacting between each pair of BtuB monomers. This would not be expected to be the case for reconstituted BtuB, but additional measurements with extended echo times in the DEER experiment will be required to make this determination and accurately resolve the distance. Finally, it is interesting to note that evidence for longer distances, like those seen for BtuB, appears for FecA expressed and double labeled in whole cells. However, unlike BtuB, the data obtained from FecA in the reconstituted system using the *dsbA* ^*−*^ strain shows no evidence for an extended distance (Figure 4c). Although additional work is needed to establish the presence and source of this distance component, this preliminary data suggests that the nature of the interactions between these OMPs may differ, and that the FecA‐FecA interaction may require components in the native outer membrane (perhaps LPS), whereas BtuB‐BtuB interactions do not.

The substrate‐dependent loop movements observed here in FecA (Figure 4b) suggest that like BtuB, conformational changes in the extracellular loops of FecA are altered by the native environment. The crystal structures of FecA (Yue et al., 2003) reveal large changes in the positions of loops 7 and 8 upon the addition of substrate. These large movements are thought to be important for the high affinity of ferric citrate to the transporter, and for effectively trapping and preventing the release of the substrate back into extracellular space. However, the data shown in Figure 4b indicate that while there is a substrate‐induced shift in loop position in cells consistent with the crystal structure, the 8–11 interloop distance seen for the apo state persists and is in fact the dominant configuration. Although more work is necessary to determine the nature of these conformations, the two populations seen by DEER likely reflect an equilibrium between the two conformational states. If this is the case, the free energy differences between these two states is small and the loops may not be functioning to trap the substrate. For BtuB, the lack of loop movements in the intact cell led to the conclusion that structural changes within the core of BtuB were responsible for tightly binding and trapping the substrate (Nyenhuis, Nilaweera, & Cafiso, 2020), and this may be the case for FecA as well. Remarkably, the FecA 8–11 loop distance does not change with substrate addition when the protein is reconstituted into POPC (Figure 4b), indicating that the structural change seen in cells is dependent upon components that are absent in the reconstituted system.

In summary, we provide evidence that the expression of an outer‐membrane protein in a strain where the Dsb system is inhibited facilitates the efficient spin labeling pairs of cysteines both in‐vivo and in vitro. For FecA, it was not possible to label the extracellular loops in cells unless a *dsbA* ^*−*^ strain was used, and measurements in cells show evidence for substrate‐dependent changes in loop structure that are not observed in a purified reconstituted phospholipid membrane. For BtuB, expression in a *dsbA* ^*−*^ strain dramatically improved the efficiency and quality of the EPR data obtained in vitro. This improved labeling efficiency also revealed the presence of intermolecular BtuB‐BtuB interactions that had not been previously observed in a reconstituted membrane environment.

## 4 MATERIALS AND METHODS

### 4.1 Cell lines and plasmids

For the vitamin B_12_ transporter, BtuB, the pAG1 plasmid with WT btuB gene and the *E. coli* strain RK5016 (*−argH, −btuB, −metE*) was obtained from Professor Robert Kadner, University of Virginia. For FecA, the pIS711 plasmid carrying the WT *fecA* gene downstream of the phage T7 gene 10 promoter, was generously provided by Volkmar Braun (University of Tübingen, Tübingen, Germany). The pTARA plasmid was purchased from Addgene (Watertown, MA), and was used to control FecA expression levels. The pTARA plasmid harbors the phage T7 RNA polymerase gene under control of the araBAD promoter. Both wild‐type and mutant *dsb E. coli* strains were obtained from the Coli Genetic Stock Center (Yale University, New Haven, CT). Strain RI89 carries the WT Dsb system (*araD139 Δ*(*araABC‐leu*)*7679 galU galK Δ*(*lac*)*X74 rpsL thi phoR Δara714 leu+*), whereas strain RI90 carries the *dsbA* null mutation system (*araD139 Δ(araABC‐leu*)*7679 galU galK Δ*(*lac*)*X74 rpsL thi phoR Δara714 leu+dsbA:: Kanr*). FecA was also expressed in the standard *E. coli* strain BL21(DE3), which lacks outer membrane porins such as OmpA, OmpC, and LamB (Prilipov et al., 1998).

### 4.2 PCR mutagenesis and OMP expression

BtuB double cysteine mutations (V90C‐T188C, D6C‐Q510C) were generated previously (Landeta et al., 2018), and FecA double cysteine mutations (Q528C‐T666C, K12C‐N256C, T70C‐N256C) were constructed using site‐directed mutagenesis and verified by DNA sequencing (Genewiz, South Plainfield, NJ). The *btuB* containing plasmids were transformed into both RK5016 and *dsbA* ^*−*^ strains and the *fecA* containing plasmids were transformed into either BL21(DE3), the *dsbA* ^*−*^ strain, or the *dsbA* ^*−*^ strain with a previously incorporated pTARA plasmid.

A single colony was used to inoculate Luria‐Bertani (LB) media and prepare a glycerol stock which was stored at −80°C. For BtuB, precultures of Minimal Media (100 mM phosphate buffer, 8 mM (NH_4_)_2_SO_4_, 2 mM sodium citrate, 100 mg/mL ampicillin, 0.2% w/v glucose, 150 mM thiamine, 3 mM MgSO_4_, 300 mM CaCl_2_, 0.01% w/v methionine, 0.01% w/v arginine) were inoculated using the glycerol stock and later used to inoculate the main culture. For FecA, a preculture of Luria‐Bertani (LB) with appropriate antibiotics was inoculated using the glycerol stock and later was used to inoculate the main culture in Minimal Media. FecA was overexpressed by induction with 0.5 mM IPTG at OD_600_ of 0.5–0.6 for 5 h at 37°C. In both cases, an initial overnight preculture growth was followed by an 8 h main culture growth. For BtuB, an initial 8 h preculture growth was also followed by an overnight main culture growth for the RK5016 strain. The pTARA mediated expression was induced at OD_600_ of 0.1 by adding arabinose up to final concentration of 0.1% w/v.

### 4.3 OM and reconstituted BtuB and FecA sample preparation

An intact OM preparation is obtained from the total cell membrane fraction using a standard procedure. This involves treating the cell membrane fraction with 1% sarkosyl to remove the inner membrane, and then pelleting the OM and removal of the sarkosyl by centrifugation. For measurements made in the OM preparation, the preparation was spin labeled at this stage (Nilaweera et al., 2019). For protein that was to be purified, the OM preparation was solubilized with OG as described previously, and 5 mL of the solubilized OM sample then was treated with 100 μL of 22 mM MTSL [(1‐Oxyl‐2,2,5,5‐tetramethylpyrroline‐3‐methyl) methanethiosulfonate] followed by incubation for 2–3 h at room temperature (RT) (Nilaweera et al., 2021). Spin labeled BtuB or FecA was purified using ion‐exchange chromatography as described previously (Coggshall et al., 2001; Fanucci et al., 2002). For some samples, BtuB was taken through two purification steps and treated with DTT following the first purification as described previously (Rassam et al., 2015; Xu et al., 2006). Briefly, the solubilized OM sample underwent an initial ion‐exchange followed by treatment of the BtuB fraction with 6 mM dithiothreitol (DTT) at RT for 30 min. The sample then underwent a buffer exchange into 25 mM Tris, 17 mM OG to remove excess DTT. These samples were then treated with 100 μL of 22 mM MTSL at RT for 2–4 h followed by a second ion‐exchange to purify BtuB from other OMPs and to remove excess MTSL (Rassam et al., 2015; Xu et al., 2006). Purified BtuB and FecA were quantified using the Bradford Assay (Bradford, 1976; Sedmak & Grossberg, 1977).

The purified and spin‐labeled BtuB or FecA samples were reconstituted into 1‐palmitoyl‐2‐oleoyl‐sn‐glycero‐3‐phosphocholine (POPC) vesicles by the addition of OG/POPC (10:1) mixed micelles to the purified BtuB or FecA samples at a protein: lipid ration of 1:25. The samples were then dialyzed against six 4 L dialysis buffer changes (10 mM HEPES, 128 mM NaCl, and 1 mM EDTA at pH 6.5) at 10–12 h intervals (Nyenhuis, Nilaweera, Niblo, et al., 2020; Rassam et al., 2015; Xu et al., 2006).

### 4.4 Whole cell spin labeling of FecA

For spin labeling FecA in cells, an identical protocol to that used for BtuB in cells was followed (Landeta et al., 2018; Nilaweera et al., 2021) where pTARA mediated expression was induced at an OD_600_ of 0.1 by adding L‐arabinose to a final concentration of 0.1% w/v. Briefly, cells were grown until early log phase and harvested at OD_600_ of 0.3. Cells were resuspended in 100 mM HEPES (pH 7.0) containing 2.5% (w/v) glucose followed by MTSL spin labelling at RT for 30 mins with a label concentration of 0.02 mg/mL as described previously (Landeta et al., 2018; Nilaweera et al., 2021). Spin labeled cells went through two washing steps in 100 mM HEPES with 2.5% (w/v) glucose to remove any excess spin label and were then immediately used for EPR.

### 4.5 EPR measurements

For CW‐EPR measurements, 5 μL sample with 1 μL dialysis buffer was loaded into 0.84 × 0.6 mm^2^ quartz capillaries (VitroCom, Mountain Lakes, NJ) and used as apo sample whereas 5 μL sample with 1 μL of either 1 mM Vitamin B_12_ or 1 mM Ferric Citrate was used as the substrate bound sample. EPR spectra were recorded at room temperature at X‐band using a Bruker EMX spectrometer (Billerica, MA) with an ER 4123D dielectric resonator using a sweep width of 100 gauss (G), a modulation amplitude of 1 G, and 2 mW incident microwave power. Data were collected as additive averages of 10 scans and were normalized by their second integral. For DEER, a 16 μL sample with 4 μL of concentrated glycerol was loaded into 1.1 × 1.6 mm^2^ quartz capillaries (VitroCom, Mountain Lakes, NJ). Either 2 μL of 1 mM Vitamin B_12_ or 2 μL of 1 mM Ferric Citrate were added for samples with substrate. All DEER experiments were performed on a Bruker E580 spectrometer operating at Q‐band (Bruker BioSpin, Billerica, MA) with the following hardware: Bruker EN5107D2 dielectric resonator, Bruker SpinJet‐AWG, and a 300 W TWT amplifier (Applied Systems Engineering, Benbrook, TX). Experiments were run at 50 K using the standard dead‐time free 4‐pulse DEER experiment, with a 10 ns π/2 pulse and 20 ns π pulses. All pulses were rectangular. The separation between observe and pump frequencies was 75 MHz. Acquisition times for most samples typically ranged from 10 to 15 h. Without arabinose induced expression of FecA, acquisition times were in excess of 48 h.

### 4.6 Data processing

All DEER data were processed with DeerAnalysis (Jeschke et al., 2006) using the DEERNet routine (Worswick et al., 2018). All raw unprocessed DEER data have been included as Supporting Information. Simulated distance distributions were generated using the software package MMM and the default rotamer library (Jeschke, 2018; Polyhach et al., 2011). Protein structure images were generated using Pymo (DeLano, 2002).

## CONFLICT OF INTEREST STATEMENT

The authors declare no conflicts of interest.

## Supporting information


**Data S1** Supporting Information.
