# Supplementary material for: A disulfide chaperone knockout facilitates spin labeling and pulse EPR spectroscopy of outer membrane transporters
Source: Protein Sci. 2023 Jul 1;32(7):e4704. doi: 10.1002/pro.4704 (PMC10288552; doi:10.1002/pro.4704)
Supplement: Supplementary file 1 — Data S1 Supporting Information. [file PRO-32-e4704-s001.docx]

Supplementary Material

A disulfide chaperone knockout facilitates spin labeling and pulse EPR spectroscopy of outer membrane transporters

Viranga W. Wimalasiri, Kinga A. Jurczak, Monika K. Wieliniec, Thushani D. Nilaweera, Robert K. Nakamoto and David S. Cafiso

This folder contains unprocessed DEER data in the Bruker Elexsys format generated by the Bruker Xepr software. These data were used to generate the fits and distributions shown in the indicated Figures.
